# The Most Comprehensive Study at Single-Cell Resolution: A Giant Step toward Understanding Gastric Cancer

**DOI:** 10.1055/s-0042-1758763

**Published:** 2022-12-08

**Authors:** Fei-Yu Diao

**Affiliations:** 1 Department of General Surgery, Sun Yat-sen Memorial Hospital, Sun Yat-sen University, Guangzhou, People's Republic of China

In 2020, gastric cancer is the fifth common cancer and the fifth leading cause of cancer death in the world. 1 It has the highest incidence and mortality rate in Asian countries, such as China, Japan, and South Korea. 2 3 Heterogeneity at the histologic, transcriptomic, genomic, and epigenomic levels exists between gastric cancer patients (interpatient heterogeneity) and within individual tumor mass (intertumoral heterogeneity). It leads to different cancer biological behaviors and treatment response. 4 Therefore, biomarkers developed based on the heterogeneity of gastric cancer play an important role in guiding clinical treatment and improving patient prognosis. 5 6 Although some current cancer genome projects, The Cancer Genome Atlas (TCGA) and Asian Cancer Research Group, have made great progress in facilitating the molecular typing of gastric cancer, their role in improving the prognosis of gastric cancer patients is limited. Therefore, to conduct high-resolution studies at molecular level in a wide range of patients to guide the clinical treatment of gastric cancer is necessary.

Previous “bulk-transcriptome” studies have found that each gastric cancer case has a unique expression profile contributed by cancer cells and resident cell types of tumor microenvironment (such as cancer-associated fibroblasts, immune cells, and endothelial cells, etc.), 7 but the underlying molecular mechanisms of how tumor microenvironment resident cells drive tumor phenotype evolution and clinical progression remain unknown. With the advances in bioinformatics, bulk sequencing data has been successfully decomposed into lineage-specific constituent programs, but this approach fails to discern rare cell populations, fine-scale tissue lineages, cell–cell interactions, and relationships between lineages. 8 Single-cell RNA sequencing (scRNA-seq) is the primary tool for addressing these issues. It can detect gene expression in thousands of cells simultaneously, enabling comprehensive analysis of different cell types in tumor mass under different conditions. Indeed, scRNA-seq on gastric cancer tissues from various sources has provided unique insights of cancer biology. However, these current scRNA-seq studies are limited by the number of samples and cells, as well as the dissociation requirements for tissues, which had led to the loss of many key information, especially spatial information. Thus, digital spatial analysis, in situ sequencing, and multiplexed error-robust fluorescence in situ hybridization platforms have been developed to maximize the preservation of spatial information, and thereby allowing the in-depth analysis of tumor–tumor microenvironment interactions.

In a study recently published in *Cancer Discovery* , titled “Single-Cell Atlas of Lineage States, Tumor Microenvironment, and Subtype-Specific Expression Programs in Gastric Cancer,” Kumar et al 9 delineated a comprehensive single-cell atlas of gastric cancer specimens across clinical stages and histologic subtypes by scRNA-seq and complemented this atlas by spatial transcriptomics, orthogonal validation in independent bulk RNA-seq cohorts, and functional demonstration using patient-derived organoids and in vivo models. This scRNA-seq study discovered several new rare cell populations undergoing state transition in gastric cancer, associations between plasma cell and cancer-associated fibroblast sublineages, and gastric cancer clinical stages or histological subtypes, and gastric cancer-associated cell type-specific expression programs. One of the strengths of this study is the large number of cells analyzed by scRNA-seq (more than 200,000 cells), which is much higher than the sum of all previous gastric cancer scRNA-seq studies. Additionally, the clinical specimens collected in this study covered multiple clinical stages and subtypes of gastric cancer and also included a comparative analysis of organoids. Most importantly, the main findings of this study are orthogonally verified by new spatial transcriptomics technology (digital spatial profiling [DSP]).

Previous scRNA-seq studies compared tumors and normal tissues in a cell-lineage-specific manner, such that their tumor profiles were composite tumor profiles assembled from different error signatures expressed by different lineages. By using DSP technology, this study found that chief cells and intestinal-type cells contributed the largest cancer-associated gene expression differences in cancer epithelial component. The most noteworthy is that the intestinal-type epithelial cells (EpiInt1) highly expressed oncogenes of gastric cancer, suggested that EpiInt1 population was a key epithelial cell population in the transition of metaplastic gastric epithelial cells into cancer cells. However, the platform used in this study is unable to detect single-cell alterations at both the DNA and RNA levels on a genome-wide scale currently, so the authors could not directly infer alterations based on single-cell DNA. In addition, this study used a global clustering approach and thus could not identify the more granular cell types that only refined local clustering approaches could distinguish. 10 By analyzing the data for non-epithelial cell types, the authors identified multiple cancer-associated fibroblast clusters. Among them, the gene expression differences between LUM ^+^ cancer-associated fibroblasts and CSPG ^+^ pericyte cancer-associated fibroblasts reflected their different functions in tumor development and progression (LUM ^+^ cancer-associated fibroblasts may promote the proliferation of gastric cancer cells, while CSPG ^+^ pericyte cancer-associated fibroblasts may be involved in angiogenesis). Additionally, this study identified a small cluster of non-epithelial cells undergoing endothelial–mesenchymal transition (a cluster of cells co-expressing PLVAP and RGS5) and confirmed the presence of this cell cluster by doublet filtering and orthogonal RNAScope analysis. These observations mentioned above were not discernible in previous RNA-seq data. They indicated that an individual cell lineage that has undergone markedly malignant transformation can trigger alterations in the entire tumor ecosystem.

Several previous studies have preliminarily explored the functions and molecular mechanisms of cancer-associated fibroblasts in gastric cancer, but they have not defined the heterogeneity of cancer-associated fibroblast. This study reported that cancer-associated fibroblasts in gastric cancer included three main subtypes (STF1, STF2, and STF3). The STF3 subtype is characterized by high expression of both FAP and INHBA. The authors confirmed the expression correlation of FAP and INHBA in multiple orthogonal settings. Subsequent functional studies demonstrated that INHBA could regulate the expression of FAP through the TGFβ signaling pathway. This finding is consistent with a recent bulk RNA-seq study that used laser capture microdissection-derived cancer-associated fibroblasts. 11 Studies in other cancer types also reported that high expression of INHBA in cancer-associated fibroblasts is associated with patient prognosis. 12 Therefore, INHBA and the downstream signaling pathways can be used as novel therapeutic targets for gastric cancer.

Lauren classification divide gastric cancer into intestinal-type, diffuse-type, and mixed-type. The intestinal-type is mainly attributed to the Correa cascade, while the evolution and the underlying molecular mechanisms of the diffuse-type remain unclear. By comparing the cell lineages in tumor microenvironment of different gastric cancer subtypes, this study observed that the number of plasma cells was significantly increased in diffuse-type gastric cancer tissues. This is consistent with the findings of a previous study based on the TCGA dataset. 13 Furthermore, increased plasma cells are strongly associated with poor response to immune checkpoint inhibitors in gastric cancer. 14 To define the mechanisms responsible for the increase in plasma cells, the authors analyzed the expression of KLF2, a transcription factor that regulates the homing of plasma cells and multiple myeloma cell adhesion, in diffuse gastric cancer. They found that KLF2 expression in diffuse-type epithelial cell clusters was positively correlated with the proportions of plasma cells, and KLF2 likely drive plasma cell recruitment through paracrine cell signaling pathways. Interestingly, the authors also found that the epithelial cells of diffuse gastric cancer could upregulate multiple immune signaling pathways to mimic immune cells. This “epithelial-immune cell state” has also been reported by other groups in other cancer types. 15 This study reported the existence of this phenomenon in diffuse-type gastric cancer for the first time, laying the foundation for exploring the underlying mechanisms of epithelial-plasma cell crosstalk in diffuse-type gastric cancer.

Patient-derived organoids have become an emerging system in cancer research platforms that can be used in precision oncology, anti-cancer drug testing, and functional exploration of cancer driver genes. This study is the largest scRNA-seq analysis of gastric cancer to date, and the patient-derived organoids in this study indeed maintained most cell types in gastric cancer tissues, making this scRNA-seq data a unique data resource. In the future, this data resource can be used to further explore new cell types and explore the mechanisms of cell–cell interactions in gastric cancers at the genetic, epigenetic, transcriptional, and spatial context levels, which will help us better understand gastric cancer.
